# Effect of nutritional supplementation of breastfeeding HIV positive mothers on maternal and child health: findings from a randomized controlled clinical trial

**DOI:** 10.1186/1471-2458-11-946

**Published:** 2011-12-22

**Authors:** Gurpreet Kindra, Anna Coutsoudis, Francesca Esposito

**Affiliations:** 1 Department of Paediatrics and Child Health, Room 257, DDMRI Building, Nelson R Mandela School of Medicine, University of KwaZulu-Natal, Congella 4013, Durban, South Africa

**Keywords:** HIV positive mothers, HIV exposed infants, breastfeeding, nutrition supplement, body composition, anthropometry, SRQ 20, RCT

## Abstract

**Background:**

It has been well established that breastfeeding is beneficial for child health, however there has been debate regarding the effect of lactation on maternal health in the presence of HIV infection and the need for nutritional supplementation in HIV positive lactating mothers.

**Aims:**

To assess the effect of nutritional supplementation to HIV infected lactating mothers on nutritional and health status of mothers and their infants.

**Methods:**

A randomized controlled clinical trial to study the impact of nutritional supplementation on breastfeeding mothers. Measurements included anthropometry; body composition indicators; CD4 count, haemoglobin and albumin; as well as incidence rates of opportunistic infections; depression and quality of life scores. Infant measurements included anthropometry, development and rates of infections.

**Results:**

The supplement made no significant impact on any maternal or infant outcomes. However in the small group of mothers with low BMI, the intake of supplement was significantly associated with preventing loss of lean body mass (1.32 kg vs. 3.17 kg; *p *= 0.026). There was no significant impact of supplementation on the infants.

**Conclusions:**

A 50 g daily nutritional supplement to breastfeeding mothers had no or limited effect on mother and child health outcomes.

**Clinical trial registration:**

ISRCTN68128332 (http://www.controlled-trials.com/ISRCTN68128332)

## Background

Breastfeeding is the optimal source of nutrition for all infants [1,2]. Although breastfeeding is associated with a risk of post-natal vertical transmission of HIV; it has been well established that in resource-poor communities, there are serious repercussions of not breastfeeding or even of a short duration breastfeeding on HIV free survival of the infants [3-8]. Studies have shown that effective antiretroviral prophylaxis to the mother or infant during breastfeeding can reduce vertical transmission to as low as 1% [7,9-12]. WHO therefore revised its PMTCT (prevention of mother to child transmission) guidelines in 2010 to help maximize HIV free survival of infants by recommending longer breastfeeding with prophylaxis as well as the timely start of antiretroviral therapy (ART) in the mothers [13].

Women in resource-constrained settings such as sub-Saharan Africa are carrying a double burden of HIV and food insecurity and are therefore prone to nutritional deficiencies [14-17].

Nutrition and immunity have a synergistic relationship and deficiencies in either can have serious health implications [18,19]. Apart from food shortages, HIV infection is also frequently associated with malnutrition due to causes such as anorexia; increased nutrient requirements due to increased metabolism; and decreased absorption due to opportunistic infections [20]. Besides this, the resting energy expenditure in HIV infected women has been shown to be higher than that in their HIV uninfected counterpart [21]. There was therefore some concern raised regarding the effect of breastfeeding on maternal health [22]. However, it was shown by Papathakis et al. that although HIV positive lactating mothers lost more weight and subcutaneous fat than the HIV negative women, there was no significant difference in their change in lean body mass [23]. Two longitudinal studies of assessments of maternal morbidity and mortality also showed that breastfeeding did not add an additional burden on the health of HIV positive mothers [24,25].

It has been estimated, based on a mean milk production of 699-854 g/day, that exclusively breastfeeding mothers require an increased energy intake of 614-750 kcal/day [26]. The energy conversion from food intake and body stores to milk energy has been shown to be very efficient at 80% [26]. Lactating women in most societies tend to have a normal to lower total energy expenditure in comparison to the non-lactating women as they are mostly sedentary; however in certain societies, mothers have to return to work in order to sustain a living especially to labour-intensive work. Well-nourished women tend to lose more weight (0.8 kg/month) during lactation in comparison to the under-nourished women (0.1 kg/month) possibly due to conservation of energy [26]. This rate of weight loss in the well-nourished women corresponds to a mobilization of energy from their stores at a rate of 170 kcal/day [26]; however it is estimated that they would still require an additional 505 kcal/day during breastfeeding; and undernourished mothers who already have low reserves need to compensate by increasing their daily energy consumption by 675 kcal/day [26]. Women in our clinic community are very food insecure due to unemployment and mostly rely on child support grants. The few who are employed usually work as domestic workers this being a peri-urban area. This obviously leads to the question 'would giving food or a well-balanced food supplement help to circumvent the energy cost of lactation?'

Since to date there are no published studies on the impact of nutritional supplements on HIV positive lactating mothers we tested the effect of a food supplement in the form of a peanut/soya milk based spread enriched with micronutrients(Table 1). The supplement, "Sibusiso ready food supplement" was also being used by the national department of health (DOH) as a therapeutic supplement in malnutrition and HIV associated wasting; and was therefore presumed to be acceptable in taste and texture to our community. Since loss in lean body mass (LBM) is a prognostic marker in HIV infection [27] and changes in body composition are more sensitive in testing interventions [28] we used change in LBM rather than anthropometric measurements alone to assess the effect of the supplement. Karnofsky score, a performance score used globally as an indicator of health and well-being and recommended for use in adults with HIV infection by WHO was also used as an indicator of health [29].

**Table 1:** Composition of Sibusiso ready food supplement

Nutrients	Per 50 g serving	%RDA per 50 g
**Energy (kj)**	1176	

**Protein (g)**	8	

**Carbohydrate (g)**	24	

**Fat (g)**	17.5	

**Fiber (g)**	0.95	

**Vitamin A (mcg RE)**	600	75

**Vitamin D (mcg)**	5	83

**Vitamin E (mg)**	15	100

**Vitamin K (mcg)**	25	

**Vitamin C (mg)**	75	100

**Vitamin B1 (mg)**	1.1	79

**Vitamin B2 (mg)**	1.1	69

**Vitamin B3 (mg)**	10	56

**Vitamin B6 (mg)**	1.3	50

**Folic acid (mcg)**	300	75

**Vitamin B12 (mcg)**	1.8	60

**Biotin (mcg)**	30	100

**Pantothenic acid (mg)**	4	80

**Calcium (mg)**	1000	91

**Phosphorus (mg)**	700	80

**Iron (mg)**	14	100

**Magnesium (mg)**	80	23

**Zinc (mg)**	5	33

**Iodine (mcg)**	150	100

**Selenium (mcg)**	55	100

**Sodium (mg)**	< 290	

**Potassium (mg)**	595	

**Copper (mcg)**	700	

Food security is one of the main issues, which affect the day to day living in the impoverished household, and alleviation of such a stressor through nutritive intervention may also impact positively on the mother's emotional disposition. We therefore monitored the mother's mental health status using the WHO self-reporting questionnaire (SRQ20), which has been validated in our population [30,31]. The mother's mental health status may positively affect the level of care she gives to her baby, thereby improving the baby's growth and development [32,33]. Therefore we also assessed the effect of the supplement on growth and development of the infant as well as incidence of opportunistic infections like diarrhoea and lower respiratory tract infections (LRTI). As all women in the study received a multivitamin supplement (national PMTCT guidelines) we were not in a position to test the impact of micronutrients on health and nutritional status independently but instead tested the impact of providing a food supplement in conjunction with the multivitamin on the nutritional status of the breastfeeding mothers as well as on the incidence of opportunistic infections and their disease progression.

## Methods

### Study population and design

The study sample consisted of HIV positive pregnant women of Zulu ethnicity attending the antenatal clinic (ANC) at Umkhumbane Community Health centre at Cato Manor. Breastfeeding mothers were randomized in a controlled clinical trial (RCT) to receive supplementation or non-nutritive household supplies. The supplement was a peanut/soya milk spread enriched with micronutrients and came packaged in plastic tubs. A daily serving of 50 g of the nutrition supplement provided 280 kcal energy and 8 g protein. The non-nutritive supplies included tea; shampoo and conditioner of equal monetary value and were therefore not considered to have an impact on nutrition. Randomisation took place by mothers removing a card pre-marked with group number from a slit in a closed box; this was done to enable them to be confident that the randomisation was unbiased. Randomisation was conducted by a study counsellor who maintained a separate record of the randomisation log. Outcomes were assessed by a separate clinician. To ensure that the clinician was blinded, the counsellors issued a brown bag containing either the nutritional supplement or the non-nutritive supplies. Both supplement and the non-nutritive supplies were packed in identical brown bags in an attempt to mask the allocation of group. The data on the randomization was collected using subject identification numbers only so as to reduce observer bias. To prevent dilution of the effect of the intervention and mothers discussing the content of their brown bags, separate clinic visit days were allocated for the two groups. To monitor adherence, a monthly register with mothers' signatures was maintained as well as a questionnaire was administeredto the mothers at every visit discussing daily intake of the supplement and any possible reasons for not taking the supplement including information on any benefits or side-effects that they attributed to the supplement. The counselors also tried to ascertain that the mothers were not sharing the supplement with members of their household by interviewing the mothers.

### Sample size calculations

The study was designed to detect a difference of ≥ 4 kg between the two groups. It was determined that a sample size of 40 in each group would achieve 90% power using a one-sided, two-sample *t*-test. The true difference between the means was assumed to be 0.00. The significance level (alpha) of the test was 0.025. The data are drawn from populations with mean weights with standard deviations of 6.70 and 3.60 kg obtained from piloting the body composition methods in the same population.

Allowing for a 30% loss to follow-up due to high mobility of the population post delivery, we estimated a sample size of 52 in each group. Then adding an additional 30% for loss of datadue to missed visits for body composition analyses or laboratory tests; we estimated a sample sizeof 64 in each group.

### Enrollment and exclusion criteria

HIV positive mothers attending the ANC were referred to the on-site MTCT Plus programme, an internationally funded programme that provided PMTCT services as well as comprehensive care and treatment for HIV infected mothers and their families. Mothers were routinely counselled on the feeding options and risks thereof were explained. Breastfeeding HIV positive mothers were referred to dedicated study counsellors who were housed in an independent building a few metres from the clinic. These study counsellors screened the mothers for study eligibility and counselled them on the study procedures. Only mothers planning to breastfeed for at least 6 months were eligible to enter the study. Mothers with advanced disease (CD4 < 200 or WHO stage 3 and 4) requiring ART and those not resident in the area were not eligible for recruitment. In addition, mothers of infants requiring specialized management and with gestation less than 36 weeks were not eligible for study enrolment. Interested mothers were pre-enrolled after a written informed consent was obtained at the next antenatal visit. Mothers were then seen at 2 weeks post-delivery and officially enrolled into the study.

### Study procedures and visits

Study assessments for both mothers and their infants were done at 2 and 6 weeks post-delivery and monthly thereafter till 6 months of age with a final study visit at 9 months. At each visit, a clinical examination and anthropometric measurements were done; along with a developmental assessment on the infants and Karnofsky scoring on the mothers. Body composition and SRQ 20 assessments were done on the mother at 2 weeks and then three monthly till 9 months. Mothers and the positive infants were assessed for any signs of disease progression; opportunistic infections; WHO disease staging; and CD4 counts. When indicated they were started on ART as per national guidelines [34].

A 24-hour dietary recall was done at 3, 6 and 9 months to assess for similarity of dietary intake in the groups to rule out any confounding for the outcome of supplementation. Infant feeding history was taken at each visit.

Infants received multivitamins and cotrimoxazole for *Pneumocystis jiroveci *pneumonia (PCP) prophylaxis from 6 weeks that was discontinued when the infant was confirmed HIV DNA PCR negative. Mothers received PCP prophylaxis if indicated.

Anthropometric measurements included weight; height; length; mid-upper arm circumference (MUAC); and triceps skinfold thickness (TSF) on both mother and infant. In addition head circumference measurements were taken on the infants. All measurements were performed in duplicate as per ISAK (International Society for the Advancement of Kinanthropometry) methods by either the principal investigator or the dietitian to reduce inter-observer variability and enhance reliability [35]. Body Mass Index (BMI) was calculated by dividing the weight of the subject by their height squared (kg/m^2^).

All women in the study were counseled and supported to practice exclusive breastfeeding for 6 months. Mothers whose infants were asymptomatic and who tested HIV negative at 6 weeks were counseled on either discontinuing breastfeeding at 6 months, or heat-treating expressed breast milk [36,37].

### Body composition

Saliva samples from mothers were collected to measure Deuterium enrichment using standardized IAEA (International Atomic Energy Agency) methodology [38]. These samples were analysed for total body water (TBW) using an FTIR (Fourier transform infra-red spectrophotometer). LBM was calculated from the TBW (73.2% of TBW). Fat mass (FM) was calculated as the difference between the body weight and the LBM.

### Laboratory methodology

Maternal bloods were done at 2 weeks and at 6 months and tests included haemoglobin; haematocrit; mean cell volume; platelet count; white cell count and lymphocyte count; total protein and albumin; and CD4 counts. Infants had a HIV DNA PCR done routinely at 6 weeks and the infants who tested positive had a repeat PCR at 10 weeks. A DNA PCR was repeated at 9 months in infants who tested negative at 6 weeks. All investigations were routine tests done at the accredited National Health Laboratory Services.

### Data preparation

Data was entered into an MS Access database (^©^MS Office 2003). It was rechecked for any missing information and entry errors. A unique subject identification number to maintain confidentiality identified mothers and infants.

To study associations/trends within categories: BMI was categorized as ≤ 24.9 kg/m^2 ^and ≥ 25 kg/m^2^. Disease progression was categorized as progression to WHO stage 3 or higher; a poor Karnofsky score was categorized as 80% or below (80% and above is associated with ability to do normal activity); and the WHO cutoff of an SRQ20 score of 8 and above (associated with depression) was used as an indicator of depression [30,31].

### Statistical methods

SPSS 15.0 for windows was used for analysis (^© ^SPSS Inc.). The two groups were compared for baseline socio-economic as well as clinical criteria to assess for similarity.

Continuous variables were checked for normality of distribution. Independent t tests and chi-squared tests were done for the continuous and categorical variables respectively. Non-parametric tests were conducted for the skewed continuous variables and medians with their inter-quartile range are reported.

Paired T tests for normally distributed variables and Wilcoxon signed rank test for skewed variables were performed to study changes over time in the laboratory parameters, anthropometric and body composition measurements.

Incidence rates and incidence rate ratios (IRR) were calculated for clinical events, opportunistic infections, Karnofsky score and SRQ 20 score using STATA 9.2 (^©^StataCorp).

As low BMI is considered to be an important prognostic marker for breastfeeding HIV positive women, all anthropometric, body composition, laboratory and disease progression parameters were analysed according to BMI category. As BMI ≥ 25 kg/m^2 ^was being used by the province as a cutoff for not being eligible for supplements in the HIV positive population and as our median BMI was 26 kg/m^2^, we categorized the BMI in our sample using the same categorization as the province: ≥ 25 kg/m^2 ^and < 25 kg/m^2^. An intention to treat analysis was conducted using generalized estimating equations (GEE) with robust estimators and an exchangeable covariance matrix to analyse the continuous variables over time controlling for socio-economic status; baseline CD4 and BMI. Generalised linear models (GLM) with Poisson distribution and log function were used to analyse the incidence rates of the opportunistic infections. The WHO *igrowup *tool using the 2007 WHO growth standards was used to assess the growth parameters [39].

### Ethical considerations

Ethical approval for the study was obtained from the Bioethics Committee of the University of KwaZulu-Natal (H081/05).

## Results

**Figure 1:**
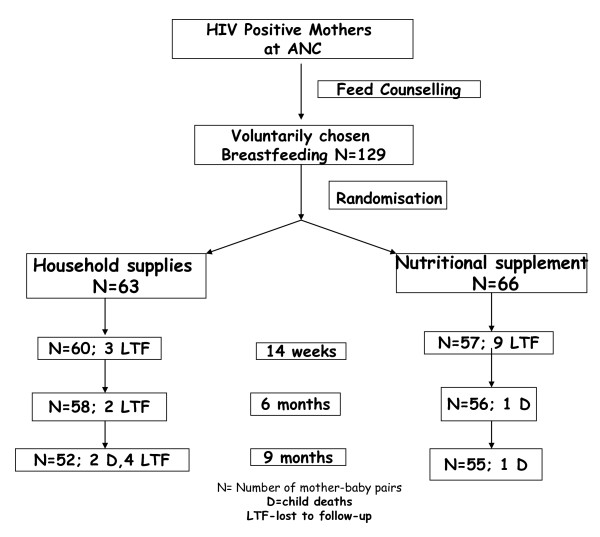
**Study design and flow diagram of follow-up**.

Between December 2006 to July 2008; 129 mothers were eligible for analysis as they fulfilled the eligibility criteria namely completing at least 2 study visits; 63 in the control group and 66 in the supplemented group (Figure 1). Study follow-up was complete by May 2009. There were no statistically significant differences between the two groups of mothers or infants at baseline (Table 2 and 3). All mothers were WHO stage 1 in the two groups except for one mother in the supplemented group who was WHO stage 2. There were no statistically significant differences between the infants at baseline.

**Table 2:** Socio-demographic and laboratory parameters of the mothers at baseline

Variables		Breastfeeding: control (n = 63)	Breastfeeding: supplemented (n = 66)	p value for the difference between groups
**Age years Mean (SD)**		24.9 (4.21)	25.85 (4.85)	0.241

**Marital status:** **Single/cohabiting (% within group)**		59 (93.7)	65 (98.5)	0.155

**Access to water** **(% within group)**	**Own tap**	36 (57.1)	32 (48.5)	0.327
		
	**Public tap**	26 (41.3)	34 (51.5)	
		
	**Other**	1(1.6)	0 (0)	

**Access to Electricity (% within group)**		38 (60.3)	30 (45.5)	0.091

**Access to** **Sanitation (% within group)**	**Waterborne**	29 (46)	28 (42.4)	0.522
		
	**Pit**	33 (52.4)	38(57.6)	
		
	**Nil**	1 (1.6)	0 (0)	

**Source of income** **(%within group)**	**Self**	12 (19)	16 (24.2)	0.286
		
	**Partner**	25 (39.7)	23 (34.8)	
		
	**Family**	21 (33.3)	26 (39.4)	
		
	**Grants**	5 (7.9)	1 (1.5)	

**Food insecurity (% within group)**		20 (31.7)	21 (31.8)	0.993

**Disclosure to family (% within group)**		29 (46)	27 (40.9)	0.557

**Disclosure to partner (%within group)**		38 (60.3)	40 (60.6)	0.973

**Contraception use (% within group)**		48 (76.2)	46 (69.7)	0.407

**Median years of schooling (IQR)**		11 (10-12)	11 (10-12)	0.515

**Median size of household (IQR)**		3 (2-5)	3 (2-5)	0.901

**Median parity (IQR)**		2 (1-3)	2 (1-3)	0.829

**Past history of TB (% within group)**		1 (1.6)	3 (4.5)	0.375

**Past history of STI (% within group)**		10 (15.9)	10 (15.2)	0.583

**BMI kg/m^2 ^Median (IQR)**		26.53 (23.37-30.1)	26.18 (23-30.67)	0.793

**CD4 cells/μL Median(IQR)**		413 (312-492)	443.5 (294.8-564)	0.658

**Haemoglobin g/dL Mean (SD)**		11.84 (1.59)	11.47 (1.52)	0.183

**Total Protein g/L Mean (SD)**		79.12 (7.12)	79.45 (8.22)	0.808

**Albumin g/L Mean (SD)**		32.27 (4.21)	30.86 (4.85)	0.065

**Table 3:** Baseline anthropometric measurements amongst the two groups of infants at 2 weeks of age

	Breastfeeding control (n = 63)	Breastfeeding supplemented (n = 66)	P value (95% CI of the difference)
**Weight kg Mean (SD)**	3.72 (0.58)	3.66 (0.48)	0.538 (-0.13, 0.24)

**Length cm Mean (SD)**	50.89 (2.42)	51.02 (1.98)	0.734 (-0.90, 0.64)

**HC cm Mean (SD)**	36.2 (1.43)	36.45 (1.19)	0.282 (-0.71, 0.21)

**MUAC cm Mean (SD)**	10.73 (0.98)	10.56 (0.93)	0.317 (-0.16, 0.50)

**TSF mm Mean (SD)**	6.67 (1.35)	6.29 (1.50)	0.135 (-0.12, 0.88)

**BMI kg/m^2 ^Mean (SD)**	14.27 (1.49)	14.03 (1.35)	0.335 (-0.25, 0.74)

**Weight/Age z scores Mean (SD)**	-0.14 (1.05)	-0.21 (0.89)	0.672 (-0.26, 0.41)

**Length/Age z score Mean (SD)**	-0.72 (1.12)	-0.63 (1.02)	0.650 (-0.46, 0.29)

**HC/Age z score Mean (SD)**	0.37 (1.06)	0.60 (1)	0.210 (-0.59, 0.13)

**BMI/Age z score Mean (SD)**	0.37 (1.09)	0.2 (1)	0.360 (-0.19, 0.53)

**Weight/Length z score Mean (SD)**	0.46 (1.07)	0.2 (1.13)	0.194 (-0.13, 0.64)

Unexpectedly, not all the mothers breastfed for the full 6 months. Mothers in the control group breastfed their infants longer than the supplemented group; median duration 6 months (range 1- 9) vs. 5.5 months (range 1-7); *p *= 0.009. Therefore, duration of breastfeeding was included as a confounder when assessing infant growth; and other maternal and infant variables. Exclusive breastfeeding rates were 58.6% at 6 months. Amongst the reasons for stopping breastfeeding before 6 months, returning to work was the most frequent (12.4%). Since the hypothesis was that the supplement was given to improve nutritional status of lactating mothers, when mothers came to their monthly visits, if they had stopped breastfeeding prior to the visit, the supplement was no longer issued. The median intake of supplement was 5.5 months (Interquartile range (IQR) 2.94- 6). While monitoring adherence, 33.3% of the mothers revealed that they did not consume the daily amount of supplement strictly according to protocol and there were occasional days when they did not use the supplement. The most common reason given was that the paste was very sweet (39.4%). When we analysed the dietary intake as assessed by 24-hour recall we found no significant differences in the dietary intakes in the two groups. The median energy intake at 3 months was 2090 kcal/day in the control group and 1972 kcal/day; however there was no significant difference between the two groups.

On assessing anthropometric and body composition measurements, no significant differences were seen between the two groups at 14 weeks or 6 months post delivery (Table 4). Sub-group analysis within each BMI category viz. ≤ 24.9 kg/m^2 ^and ≥ 25 kg/m^2 ^revealed no significant differences in the changes of the measurements except for LBM (Table 5). LBM decreased in both groups of subjects with low BMI, however there was a statistically significant lower mean decrease in the supplemented group (1.32 kg) when compared to the control group (3.17 kg); *p *= 0.026. However, in the subjects with high BMI, there were no significant differences between the groups. Further analysis using GEE adjusted for baseline age, CD4 and duration of breastfeeding was conducted. Data were analysed separately for mothers with BMI ≤ 24.9 kg/m^2 ^and ≥ 25 kg/m^2^. In the mothers with BMI ≥ 25 kg/m^2^, no effect of supplementation on any of the measurements was seen. In mothers with BMI ≤ 24.9 kg/m^2^; no significant effect of the supplementation was seen except in LBM where the supplemented group had a significantly lower loss in LBM (0.098 kg) compared to the control group.

**Table 4:** Effect of nutritional supplementation on Body composition and anthropometric measures of breastfeeding mothers from 2 weeks to 6 months

Mean change in variable over 6 months (SD)	Breastfeeding Control (n = 57)	Breastfeeding Supplemented (n = 56)	P value for the difference between the groups (95% CI for the difference)
**Weight in kg**	-0.43 (4.78)	-1.61 (4.89)	0.196 (-0.62, 2.97)

**BMI in kg/m^2^**	-0.13 (1.94)	-0.64 (1.97)	0.164 (-0.21, 1.24)

**MUAC in cm**	1.20 (1.62)	-0.90 (1.73)	0.349 (-0.33, 0.92)

**TSF in mm**	4.14 (4.69)	2.87 (4.88)	0.161 (-0.51, 3.05)

**LBM in kg**	-3.78 (4.1)	-3.20 (3.52)	0.569 (-2.59, 1.44)

**Fat mass in kg**	4.14 (4.57)	3.21 (5.77)	0.490 (-1.76, 3.63)

**%Fat mass**	6.07 (5.46)	4.5 (6.53)	0.316 (-1.55, 4.70)

**Table 5:** Sub-group analysis of the effect of nutritional supplementation on Body composition and anthropometric measures of breastfeeding mothers (by BMI category)

Change in variable over 6 months	Low to normal BMI (≤ 24.99 kg/m^2^)			High BMI (≥ 25 kg/m^2^)		
	
	Breastfeeding Control (n = 22)	Breastfeeding Supplemented (n = 27)	P value for the difference between the groups (95% CI)	Breastfeeding Control (n = 35)	Breastfeeding Supplemented (n = 30)	p value for the difference between the groups (95% CI)
**Wt kg Mean (SD)**	0.3 (4.45)	-0.94 (3.9)	0.306 (-1.17, 3.64)	-0.89 (4.99)	-2.22(5.63)	0.318 (-1.31, 3.96)

**BMI kg/m^2 ^Mean (SD)**	0.23 (1.76)	-0.36 (1.51)	0.215 (-0.35, 1.53)	-0.36 (2.04)	-0.90 (2.31)	0.316 (-0.53, 1.62)

**MUAC cm** **Mean (SD)**	1.44(1.61)	0.69(1.35)	0.083 (-0.10, 1.60)	1.05 (1.64)	1.10 (2.03)	0.910 (- 0.97, 0.86)

**TSF mm Mean** **(SD)**	4.45(4.15)	3.11(4.53)	0.291 (-1.18, 3.86)	3.95(5.04)	2.65(5.26)	0.318 (-1.28, 3.88)

**LBM kg** **Mean (SD)**	-3.71 (2.76)	-1.32(2.46)	0.026 (-4.46,-0.32)	-3.84(5.2)	-4.71(3.57)	0.597 (-2.44, 4.16)

**FM kg** **Mean (SD)**	4.58 (3.86)	1.72(4.7)	0.090 (-0.47, 6.18)	3.71(5.28)	4.4 (6.4)	0.747 (-4.98, 3.62)

**%FM** **Mean (SD)**	7.17(4.87)	2.55(5.82)	0.031 (0.46, 8.77)	4.98 (5.93)	6.05 (6.84)	0.643 (-5.77, 3.62)

There were no significant differences between the changes for all the laboratory parameters viz. CD4 count, haematology and albumin in both groups between 2 weeks and 6 months. The sub-group analyses within BMI categories ≤ 24.9 kg/m^2 ^and ≥ 25 kg/m^2 ^also showed no statistically significant differences.

There were no significant differences in the crude incidence rates or IRR of opportunistic infections such as LRTI, diarrhea, candidiasis, TB as well as the depression and Karnofsky scores between the two groups of mothers. This was confirmed on further analysis using GLM adjusting for CD4, age and duration of breastfeeding.

On assessing the effect of maternal supplementation on infant growth cross-sectionally, the MUAC, TSF, BMI measurements as well as the mean z scores for weight/age, weight/length, MUAC/age and TSF/age were significantly higher in the control group compared to the supplemented group at 14 weeks (Table 6). However, analysing infant growth longitudinally using GEE adjusted for mother's CD4 count, BMI and duration of breastfeeding these differences were no longer significant.

**Table 6:** Effect of maternal supplementation on growth of the breastfed infant

Growth and development parameters		Breastfeeding control (n = 63)	Breastfeeding supplemented (n = 66)	p value for the difference between the groups (95% CI of the difference)	*Adjusted p value for the difference between the groups (95% CI of the difference)
**Weight/age z scores Mean (SD)**	**14 weeks**	0.44 (1.03)	0.06 (1.09)	0.058 (-0.01, 0.77)	0.455 (-1.52, 0.68)
		
	**6 months**	0.61 (1.17)	0.15 (1.13)	0.035 (0.03, 0.88)	
		
	**9 months**	0.65 (1.16)	0.28 (1.15)	0.098 (-0.07, 0.82)	

**Length/age z score Mean (SD)**	**14 weeks**	-0.59 (1.08)	-0.48 (1.12)	0.613 (-0.51, 0.3)	0.891 (-1.29. 1.12)
		
	**6 months**	-0.35 (1.03)	-0.47 (1.24)	0.574 (-0.3, 0.54)	
		
	**9 months**	0.39 (1.14)	0.41 (1.17)	0.899 (-0.42, 0.47)	

**Head circumference/age z score Mean (SD)**	**14 weeks**	0.50 (1.07)	0.73 (1.1)	0.249 (-0.63, 0.17)	0.247 (-1.45, 0.37)
		
	**6 months**	0.42 (1.19)	0.66 (1.03)	0.267 (-0.64, 0.18)	
		
	**9 months**	0.51 (1.02)	0.68 (1.01)	0.368 (-0.57, 0.21)	

**BMI/age z score Mean (SD)**	**14 weeks**	1.06 (0.98)	0.46 (1.11)	0.058 (-0.01, 0.77)	0.154 (-1.29, 0.21)
		
	**6 months**	1.07 (1.14)	0.56 (1.2)	0.021 (0.08, 0.94)	
		
	**9 months**	1.17 (1.17)	0.69 (1.23)	0.042 (0.02, 0.94)	

**Weight/length z score Mean (SD)**	**14 Weeks**	1.26 (0.96)	0.64 (1.1)	0.002 (0.23, 0.99)	0.154 (-1.08, 0.17)
		
	**6 months**	1.15 (1.11)	0.67 (1.16)	0.026 (0.06, 0.9)	
		
	**9 months**	1.18 (1.14)	0.72 (1.19)	0.041 (0.02, 0.92)	

**MUAC/age z score Mean (SD)**	**14 weeks**	0.49 (0.97)	0.04 (1.08)	0.02 (0.07, 0.83)	0.145 (-4.78, 0.71)
		
	**6 months**	0.62 (1.07)	0.25 (1.17)	0.076 (-0.04, 0.79)	
		
	**9 months**	0.79 (1.05)	0.38 (1.05)	0.046 (0.01, 0.82)	

**TSF/age z score Mean (SD)**	**14 weeks**	0.64 (0.77)	0.06 (1.08)	0.001 (0.24, 0.93)	0.178 (-4.33, 0.8)
		
	**6 months**	0.95 (0.48-1.6)	0.52 (-0.09-1.23)	0.013	
		
	**9 months**	1.32 (0.47-1.75)	0.74 (-0.07-1.3)	0.004	

*Adjusted for maternal CD4 count and BMI at baseline and duration of breastfeeding

On assessing for incidence of opportunistic infections and development scores amongst the infants, no significant differences were seen between the two groups.

There were four infant deaths (3.1%). All were HIV positive and the deaths were attributed to gastroenteritis related morbidity. All HIV positive infants were started on ART.

## Discussion

There were no significant differences between the two groups at baseline in terms of socio-demographic variables and laboratory parameters. Statistically there was a difference in breastfeeding duration between the groups with the control group breastfeeding for a median of 6 months compared to 5.5 months in the intervention group, however "clinically" this difference is not really meaningful. Nevertheless we did control for breastfeeding duration in our analyses. Unexpectedly we found that not all the mothers consumed the supplement appropriately (taking the prescribed amount daily). It had been assumed that the supplement was acceptable in our community as the provincial heath department was using it in hospitals and clinics. However on asking mothers why they did not take the supplement appropriately we discovered that 39.4% of the mothers reported that the supplement was too sweet. This, together with the fact that not all mothers breastfed for the full 6 months was an obvious limitation of the study as the hypothesis of the study was that approximately 6 months of supplement would have an impact on the body composition of the mothers. Nutritional supplements in the context of HIV in South Africa are often viewed suspiciously by the population as they view carrying parcels home could direct attention towards them as HIV is still associated with stigma. This was reported by Doherty et al. when they assessed the acceptability of the free formula being provided nationally in South Africa [40]. However other studies, one in an HIV infected population and another where the HIV status was unknown found supplements to be acceptable [41,42]. A Mexican study found the acceptability of supplements to be poor due to the taste, the women preferred the tablets [43]. They also advise the use of locally available nutritious foods which would be culturally acceptable.

Davies et al. successfully showed in their "breaking the cycle project" that by training peers from a targeted community in nutrition education as well as in using culturally acceptable recipes helped them to engage with the community [44]. This led to a significant change in dietary habits. The importance of including a "holistic food based approach" using nutritionally valuable traditional food, which can be grown easily, be sustainable and more empowering is supported by the findings of our study [45].

When considered as a whole the two groups were similar in terms of changes in anthropometric measurements and body composition changes. We investigated the effect of supplementation as per BMI category to assess if the mothers on whom lactation would be an extra metabolic demand i.e. the mothers with a low BMI were not being more compromised. It was noted that the supplemented mothers with a low BMI actually lost a significantly lower amount of LBM compared to the control mothers (1.32 kg vs. 3.17 kg; *p *= 0.026). Even when controlling for baseline age, CD4 and duration of breastfeeding, this effect remained. The nutritional supplement to the group of lactating mothers with low BMI was therefore effective in preventing some loss of LBM presumably by providing additional protein to compensate for low dietary protein intake. This has been the first published study assessing the impact of nutritional supplementation on changes in LBM of HIV positive breastfeeding mothers. Prentice et al. observed increased subcutaneous fat and increased weights in their study of nutritional supplementation of non-HIV infected breastfeeding mothers; however no body composition measurements were done [46].

The nutritional supplement had no impact on any of the laboratory parameters assessed. This however was also observed in a Cochrane review of nutritional interventions in HIV infected men and non-postpartum women [47]. There were no differences between the groups for incidence rates of any of the opportunistic infections.

In view of the mean BMI being high, all mothers should be encouraged to exercise in the post-natal period as it has been shown to increase lean body mass as well as improve cardio-respiratory fitness [48]; this is especially important as HIV has been shown to be independently associated with cardiovascular disease and metabolic disorders [49].

On comparing infant growth, the control group appeared to have significantly higher mean MUAC, TSF and BMI measurements. However, on further assessment controlling for important confounders' viz. baseline maternal CD4 count, BMI and duration of breastfeeding these were no longer significant. Furthermore nutritional supplementation to the mothers had no impact on infections in their infants.

Four of the infants died; all of whom were HIV infected. The deaths were all secondary to gastroenteritis; and occurred within a few months of breastfeeding cessation (three stopped before 3 months and one at 6 months). Therefore our findings endorse the importance of the revised WHO recommendations which encourage breastfeeding for 12 months (in the presence of ARV prophylaxis) because they have taken into account not only HIV infection but also child survival [13].

### Study limitations

The study was powered to detect a 4 kg difference in lean body mass between the supplemented and non-supplemented groups. Although this may have been a bit too ambitious we particularly chose this effect as we wanted to see a more definitive impact because of the huge cost implications if such an intervention was to be instituted as a policy following this study. This study was therefore limited in that it obviously did not have sufficient power to detect a a smaller difference in lean body mass as well as any effect on CD4 counts and other parameters.

The limitations imposed by the slightly less than expected adherence to the supplement have already been discussed; nevertheless the study was planned as an "intention-to-treat" study. A further limitation of the study could be that the supplementation was not given as a meal replacement but as a supplement at a dose of 50 g/day; which only covered a fraction of the macronutrient requirements. We did however compare the dietary intake of the two groups of breastfeeding mothers which we found to be similar therefore the impact of the supplement was not influenced by background dietery intake.

Further, we had not determined the categorisation of BMI at the onset of the study, although WHO definitions were used to determine the cutoff.

## Conclusions

Nutritional supplementation had no impact on the group of mothers as a whole except for the small group of mothers with low BMI. There is however a possibility that if a larger daily dose of the supplement was used instead of 50 g daily, an impact of the intervention on prognostic indicators may have been seen. We would therefore recommend that nutritional supplements should only be provided in a well thought-out targeted approach.

Supplement interventions often do not make an impact due to nonadherence. Nonadherence can be due to exchanging, selling and sharing the supplement or even discarding it if associated by the subject with any socially stigmatizing disease. Therefore programmes using supplement interventions should take the time to do preliminary work on acceptability of the proposed intervention.

Although there is a place for food parcels in emergency situation such as environmental crises, civil wars etc., their use in routine practice especially in today's environment of global economic instability is not sustainable and is disempowering. There is a need to put people in control of their own health. One of the solutions could be to include simple and easy to follow nutrition guidelines during health education in primary health care clinics and antenatal clinics.

Future research priorities should include studying the impact of using locally available and thus sustainable nutrient rich foods on breastfeeding mothers with a low BMI as well as the effect of nutrition education during the antenatal as well as in the well-baby clinic follow-up period which would help both mothers with a low and high BMI.

## Abbreviations

95% CI: 95% Confidence intervals; AIDS: Acquired immunodeficiency syndrome; ART: Antiretroviral therapy; BMI: Body mass index; FM: Fat mass; HIV: Human immunodeficiency virus type 1 (HIV has been used synonymously with HIV-1); ISAK: The international society for the advancement of kinanthropometry; LBM: Lean body mass; LRTI: Lower respiratory tract infection; MTCT: Mother to child transmission; MUAC: Mid-upper arm circumference; NHLS: National Health Laboratory Services; PCR: Polymerase chain reaction; PMTCT: Prevention of mother to child transmission; DNA: Deoxyribonucleic acid; SANAS: South African National Accreditation System; SRQ20: Self reporting questionnaire of 20 questions; WHO: World Health Organization

## Competing interests

The authors declare that they have no competing interests.

## Authors' contributions

GK proposed and carried out the study; did the analysis; and wrote the 1st draft. AC assisted with the study design and supervised the study, read the first draft and edited it. FE assisted with study procedures and diet analysis and read the first draft and made suggestions. All authors read and approved the final manuscript.

## Pre-publication history

The pre-publication history for this paper can be accessed here:

http://www.biomedcentral.com/1471-2458/11/946/prepub
